# Supplementary material for: Factors Influencing False-Negative Results of QuantiFERON-TB Gold In-Tube (QFT-GIT) in Active Tuberculosis and the Desirability of Resetting Cutoffs for Different Populations: A Retrospective Study
Source: Trop Med Infect Dis. 2022 Sep 30;7(10):278. doi: 10.3390/tropicalmed7100278 (PMC9607193; doi:10.3390/tropicalmed7100278)
Supplement: Supplementary file 1 [file tropicalmed-07-00278-s001.zip › Supplementary Materials Figure S1.pdf]

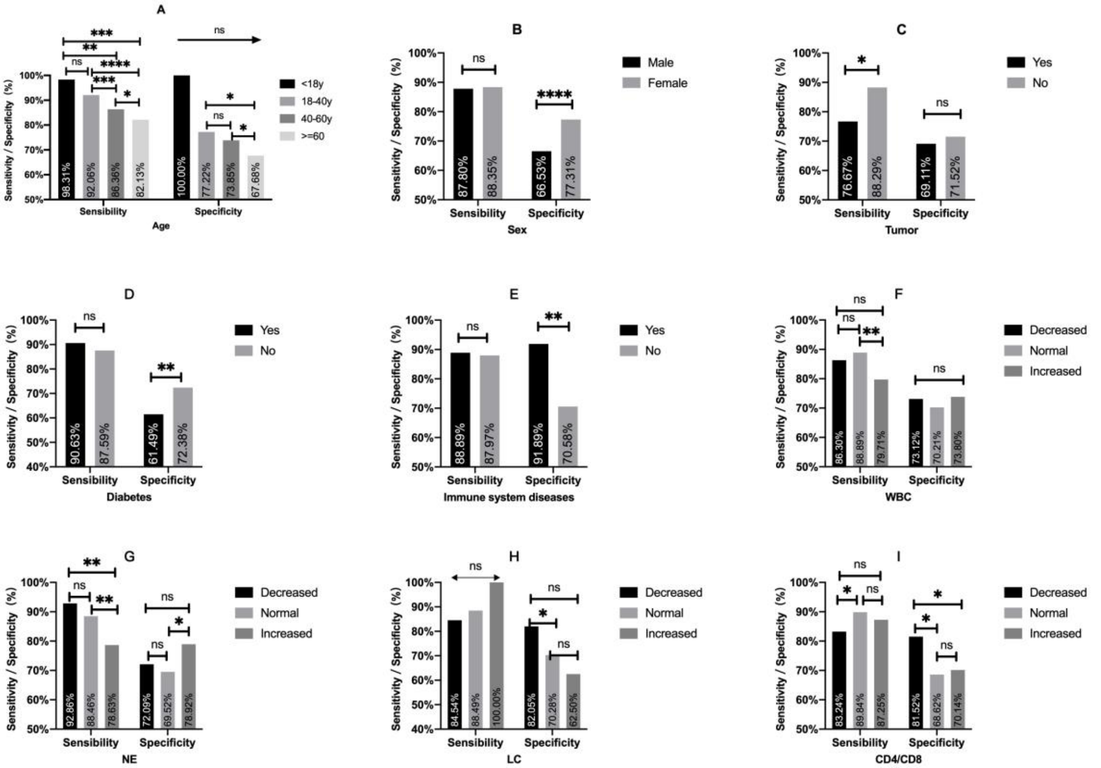

Supplementary Materials **Figure S1. Comparison of sensitivity (true-positive) and specificity (true-negative) of QFT-GIT by each influencing factor.** The comparison of sensitivity specificity between groups was represented by histogram, Statistical significance of the differences between groups were assessed by variance analysis, *P < 0.05, **P < 0.01, ***P < 0.001, ****P < 0.0001.
