# Supplementary material for: Factors Influencing False-Negative Results of QuantiFERON-TB Gold In-Tube (QFT-GIT) in Active Tuberculosis and the Desirability of Resetting Cutoffs for Different Populations: A Retrospective Study
Source: Trop Med Infect Dis. 2022 Sep 30;7(10):278. doi: 10.3390/tropicalmed7100278 (PMC9607193; doi:10.3390/tropicalmed7100278)
Supplement: Supplementary file 1 [file tropicalmed-07-00278-s001.zip › Supplementary Materials Tables S1 and S2.pdf]

Supplementary Materials Table S1 Single-factor risk analysis of false-negative results of QFT-GIT in the diagnosis of bacterial-negative tuberculosis

Influencing factor	Group	True Positive n (%)	False Negative n (%)	OR (95% CI)	*p*
Age	<18 y	1 (100)	0 (0)	0.092 (0.012-0.679)	0.019
	18-40 y	642 (91.5)	58 (8.5)	0.357 (0.246-0.520)	<0.001
	40-60 y	338 (83.3)	68 (16.7)	0.758 (0.525-1.094)	0.138
	≥60 y	275 (79.0)	73 (21.0)	Contrast	
Tumor	Yes	32 (76.2)	10 (23.8)	0.495 (0.239-1.022)	0.057
	No	1223 (86.6)	189 (13.4)		
Culture	Positive	741 (91.1)	72 (8.9)	2.543 (1.865-3.468)	<0.001
	Negative	514 (80.2)	127 (19.8)		
Site of TB	intrapulmonary	1505 (86.2)	168 (13.8)	1.378 (0.824-2.305)	0.222
	EPTB	50 (79.4)	13 (20.6)	2.239 (1.025-4.891)	0.043

	Merge	155 (89.6)	18 (10.4)	Contrast	
	Negative	562 (79.6)	144 (20.4)	Contrast	
WBC	Decreased	105 (86.8)	16 (13.2)	1.035 (0.596-1.799)	0.902
	Increased	47 (79.6)	16 (25.4)	2.313 (1.280-4.180)	0.005
	Normal	1103 (86.9)	166 (13.1)	Contrast	
NE	Decreased	74 (91.4)	7 (8.6)	0.629 (0.285-1.388)	0.251
	Increased	35 (70)	15 (30)	2.848 (1.522-5.328)	0.001
	Normal	1092 (86.3)	173 (13.7)	Contrast	
LC	Decreased	116 (88.5)	15 (11.5)	0.816 (0.466-1.431)	0.478
	Increased	4 (100)	0 (0)	1.184E-8	<0.001
	Normal	356 (89.7)	41 (10.3)	Contrast	
CD8	Decreased	494 (84.7)	89 (15.3)	1.564 (1.055-2.320)	0.026
	Increased	159 (84.6)	29 (15.4)	1.584 (0.950-2.640)	0.078
CD4/CD8	Normal	549 (88.1)	74 (11.9)	Contrast	
	Decreased	81 (81)	19 (19)	1.740 (0.999-3.033)	0.051

	Increased	379 (85.2)	66 (14.8)	1.292 (0.904-1.846)	0.159

Supplementary Materials Table S2. Single-factor risk analysis of false-positive results of QFT-GIT in the diagnosis of bacterial-negative tuberculosis

Influencing factor	Group	True Negative n (%)	False Positive n (%)	OR (95% CI)	*p*
Age	<18 y	6 (100)	1 (0)	2.612E-9	<0.001
	18-40 y	115 (77.7)	33 (22.3)	0.569 (0.373-0.866)	0.008
	40-60 y	333 (73.8)	118 (26.2)	0.702 (0.538-0.917)	0.009
	≥60 y	424 (66.5)	214 (33.5)	Contrast	
Sex	Male	473 (66.0)	244 (34)	1.727 (1.338-2.228)	<0.001
	Female	405 (77.0)	121 (23)		
Diabetes	Yes	89 (61.0)	57 (39)	0.610 (0.426-0.872)	0.007
	No	789 (71.9)	308 (28.1)		
Immune system	Yes	32 (91.4)	3 (8.6)	4.564 (1.389-15.000)	0.012
	No	846 (70.0)	362 (30.0)		

diseases					
NE	Normal	615 (69.0)	276 (31.0)	Contrast	
	Decreased	57 (70.4)	24 (29.6)	0.938 (0.570-1.543)	0.802
	Increased	128 (78.5)	35 (21.5)	0.609 (0.408-0.909)	0.015
LC	Normal	739 (69.8)	319 (30.2)	Contrast	
	Decreased	56 (81.2)	13 (18.8)	0.538 (0.290-0.997)	0.049
	Increased	5 (62.5)	3 (37.5)	1.390 (0.330-5.851)	0.653
CD4	Normal	238 (66.1)	122 (33.9)	Contrast	
	Decreased	149 (74.5)	51 (25.5)	0.668 (0.454-0.982)	0.040
	Increased	300 (71.1)	122 (28.9)	0.793 (0.586-1.074)	0.134
CD4/CD8	Normal	331 (67.3)	161 (32.7)	Contrast	
	Decreased	72 (82.8)	15 (17.2)	0.428 (0.238-0.771)	0.005
	Increased	284 (70.5)	119 (29.5)	0.861 (0.648-1.146)	0.305
